# Role of the *Citrus sinensis* RNA deadenylase CsCAF1 in citrus canker resistance

**DOI:** 10.1111/mpp.12815

**Published:** 2019-05-21

**Authors:** Hugo Massayoshi Shimo, Carolina Terassi, Caio Cesar Lima Silva, Jackeline de Lima Zanella, Gustavo Fernando Mercaldi, Silvana Aparecida Rocco, Celso Eduardo Benedetti

**Affiliations:** ^1^ Brazilian Biosciences National Laboratory (LNBio) Brazilian Center for Research in Energy and Materials (CNPEM) CEP 13083‐100 Campinas SP Brazil

**Keywords:** CCR4‐NOT‐associated factor, citrus canker, *Citrus sinensis*, CsCAF1, *CsLOB1*, PthA4, TAL effectors, RNA deadenylase activity, *Xanthomonas citri*, *Xanthomonas aurantifolii*

## Abstract

Poly(A) tail shortening is a critical step in messenger RNA (mRNA) decay and control of gene expression. The carbon catabolite repressor 4 (CCR4)‐associated factor 1 (CAF1) component of the CCR4‐NOT deadenylase complex plays an essential role in mRNA deadenylation in most eukaryotes. However, while CAF1 has been extensively investigated in yeast and animals, its role in plants remains largely unknown. Here, we show that the *Citrus sinensis* CAF1 (CsCAF1) is a magnesium‐dependent deadenylase implicated in resistance against the citrus canker bacteria *Xanthomonas citri*. CsCAF1 interacted with proteins of the CCR4‐NOT complex, including CsVIP2, a NOT2 homologue, translin‐associated factor X (CsTRAX) and the poly(A)‐binding proteins CsPABPN and CsPABPC. CsCAF1 also interacted with PthA4, the main *X. citri* effector required for citrus canker elicitation. We also present evidence suggesting that PthA4 inhibits CsCAF1 deadenylase activity *in vitro* and stabilizes the mRNA encoded by the citrus canker susceptibility gene *CsLOB1*, which is transcriptionally activated by PthA4 during canker formation. Moreover, we show that an inhibitor of CsCAF1 deadenylase activity significantly enhanced canker development, despite causing a reduction in PthA4‐dependent *CsLOB1* transcription. These results thus link CsCAF1 with canker development and PthA4‐dependent transcription in citrus plants.

## Introduction

Polyadenylation of the 3′ end of messenger RNAs (mRNAs) is a coordinated RNA modification process that plays fundamental roles not only in mRNA transport, stability and processing, but also in translational control (Fasken *et al.*, 2008; Millevoi and Vagner, 2010; Weill *et al.*, 2012). The reverse process, known as deadenylation of the mRNA poly(A) tail, also represents a critical translational control mechanism and it is regarded as the first step in mRNA decay (Temme *et al.*, 2014).

In most eukaryotic cells, the CCR4‐NOT complex is the major multi‐subunit and multi‐functional protein complex that presents mRNA deadenylase activity. The CCR4‐NOT complex was first identified in yeast, and its deadenylase activity is provided by two of its components, carbon catabolite repressor 4 (CCR4) and CCR4‐associated factor 1 (CAF1), also known as POP2 (Bai *et al.*, 1999; Basquin *et al.*, 2012; Liu *et al.*, 1998; Tucker *et al.*, 2001).

CAF1 belongs to the DEDDh subfamily of magnesium‐dependent nucleases, which have an RNase D domain required for the 3′–5′ deadenylation activity (Daugeron *et al.*, 2001; Jonstrup *et al.*, 2007; Thore *et al.*, 2003). Besides its role in mRNA deadenylation and decay, the CCR4‐NOT complex has been implicated in a variety of cellular processes, including micro RNA (miRNA)‐mediated gene silencing, transcriptional elongation and DNA repair (Collart, 2016; Denis *et al.*, 2001; Fabian and Sonenberg, 2012; Gaillard *et al.*, 2009; Kruk *et al.*, 2011). For instance, the CCR4‐NOT complex physically interacted with RNA polymerase (Pol) II and promoted transcription elongation particularly from arrested Pol II, and this interaction is in part mediated by the CCR4 and CAF1 subunits (Dutta *et al.*, 2015; Kruk *et al.*, 2011; Reese, 2013). The miRNA‐mediated deadenylation, on the other hand, required CAF1 deadenylase activity and its interaction with poly(A)‐binding proteins (PABPs) (Behm‐Ansmant *et al.*, 2006; Fabian *et al.*, 2009; Flamand *et al.*, 2016; Piao *et al.*, 2010). Importantly, recent studies in yeast using reconstituted CCR4‐NOT complex have revealed that while CCR4 is a general deadenylase that degrades PABP1‐bound poly(A) tails, CAF1 is required for the selective deadenylation of transcripts not bound by PABP1 and with lower rates of translation elongation (Webster *et al.*, 2018; Yi *et al.*, 2018). Thus, as new evidence emerges, the CCR4‐NOT complex has been regarded as a macromolecular structure that not only connects transcription to translation, but also determines the translational capacity of the cell during transcription elongation (Babbarwal *et al.*, 2014; Gupta *et al.*, 2016; Villanyi *et al.*, 2014; Webster *et al.*, 2018; Yi *et al.*, 2018).

Orthologues of the yeast CAF1 and NOT proteins have now been identified in virtually all eukaryotes (Dai *et al.*, 2016; Dupressoir *et al.*, 2001; Nousch *et al.*, 2013; Temme *et al.*, 2004; Winkler and Balasco, 2013). Nevertheless, in contrast to yeast and animals, the role played by CAF1 proteins in plants is less clear.

The *Capsicum annuum CAF1* gene was the first to be identified as a gene upregulated in response to *Xanthomonas axonopodis* pv. *glycines* infection (Lee *et al.*, 2004). Overexpression of *CAF1* in tomato plants resulted in enhanced resistance against *Phytophthora infestans* whereas its down‐regulation enhanced susceptibility to the pepper bacterial spot pathogen *X. axonopodis* pv. *vesicatoria* (Sarowar *et al.*, 2007). Similarly, overexpression of the *Arabidopsis thaliana AtCAF1a* and *AtCAF1b* genes led to an increase in the expression of pathogenesis‐related protein (PR) genes and enhanced resistance against *Pseudomonas syringae* pv. *tomato*. Conversely, down‐regulation of *AtCAF1a* and *AtCAF1b* resulted in reduced expression of PR proteins and increased susceptibility to *P. syringae* pv. *tomato* (Liang *et al.*, 2009).

CAF1 proteins have also been implicated in the control of plant development and stress responses. Tomato plants overexpressing CAF1 showed enlarged leaf cells whereas plants silenced for CAF1 showed reduced growth (Sarowar *et al.*, 2007). In *Arabidopsis* and rice, CAF1 proteins are induced by multiple stress‐related hormones and types of stress including drought, cold and wounding (Chou *et al.*, 2014; Liang *et al.*, 2009; Walley *et al.*, 2010). These results thus indicate that, in plants, CAF1 proteins play important roles in cell growth, stress responses and defence against microbial pathogens.

Previously, we have identified a citrus CAF1 homologue gene, *CsCAF1*, that was up‐regulated in sweet orange (*Citrus sinensis*) leaves in response to infection by the citrus canker pathogen *Xanthomonas aurantifolii* pathotype C (Xa), a *Xanthomonas citri* (Xc)‐related bacterium that causes canker in Mexican limes but a defence response in sweet oranges (Abe and Benedetti, 2016; Cernadas *et al.*, 2008). Since *CsCAF1* was induced in the hypersensitivity response triggered by Xa in sweet orange, we hypothesized that it might play a role in the defence against citrus canker bacteria (Cernadas *et al.*, 2008).

Here, we confirm that *CsCAF1* expression correlates with the defence response induced by Xa in sweet orange leaves and show that the protein encoded by the *CsCAF1* gene, CsCAF1, displays a magnesium‐dependent 3′–5′ RNA deadenylase activity. In addition, we show that CsCAF1 interacted with four citrus proteins associated with the CCR4‐NOT complex, and with PthA4, the main Xc transcriptional activator‐like (TAL) effector required for canker formation and transcriptional activation of the citrus canker susceptibility gene *Lateral Organ Boundaries 1*, *CsLOB1* (Abe and Benedetti, 2016; Hu *et al.*, 2014; Pereira *et al.*, 2014; de Souza *et al.*, 2012). We also present evidence suggesting that PthA4 inhibits CsCAF1 deadenylase activity and stabilizes the *CsLOB1* mRNA in Xc‐infected leaves. Our data suggest that by targeting the CCR4‐NOT complex, the effector protein PthA4 enhances transcription and translation of *CsLOB1* to promote cell hypertrophy and hyperplasia in citrus. Consistent with this idea, a novel adenine analogue inhibitor of CsCAF1 significantly enhanced canker development in Xc‐infected plants, suggesting that CsCAF1 restricts cell growth in citrus.

## Results

### Increased CsCAF1 expression correlates with defence against * Xanthomonas* infection

**Figure 1:**
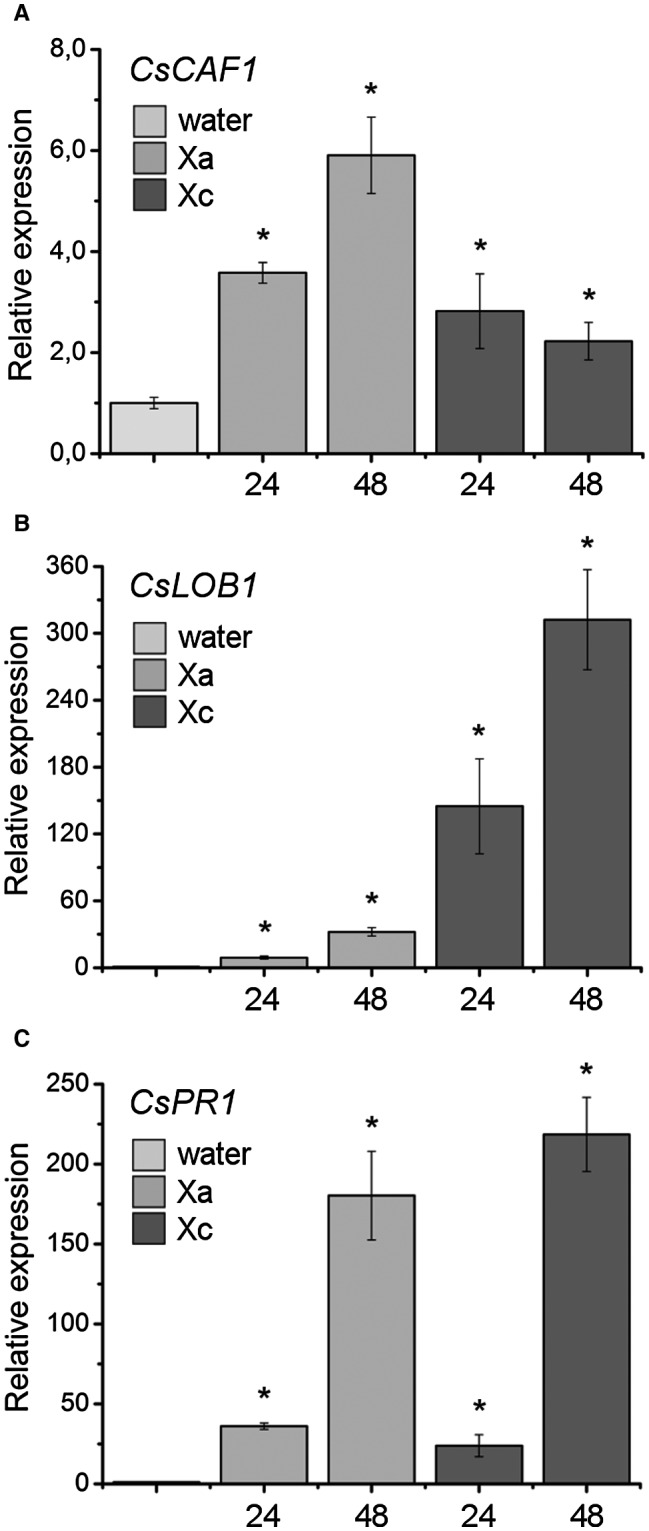
*CsCAF1* expression is increased in the incompatible interaction. qPCR analyses showing the expression levels of *CsCAF1* (A), *CsLOB1* (B) and *CsPR1* (C) in sweet orange leaves inoculated with Xa or Xc, relative to water infiltrations used as control treatments (fold change 1.0), at 24 and 48 h after bacterial inoculation. While *CsCAF1* is preferentially induced in the incompatible interaction, *CsLOB1* is highly induced by Xc. The expression of *CsPR1*, a marker gene for Xc and Xa infection, is significantly increased at 24 and 48 h after bacterial inoculation. Values are the means of triplicates of three independent biological samples. Error bars represent the standard deviations and asterisks denote statistically significant differences at the 0.05 level, relative to control (water).

Previous large‐scale gene expression analysis revealed that the *CsCAF1* gene (XP_006481524.1) was up‐regulated in the incompatible interaction of sweet orange plants infected with Xa (Cernadas *et al.*, 2008). To confirm these results, sweet orange leaves were infiltrated with Xa, Xc or water, as control, and the expression of *CsCAF1* was monitored by quantitative RT‐PCR. The expression levels of *CsLOB1*, a direct target of PthA4, and the citrus pathogenesis‐related *PR1*, a marker gene for Xa and Xc infection (Abe and Benedetti, 2016; Cernadas *et al.*, 2008; Hu *et al.*, 2014; Pereira *et al.*, 2014), were also evaluated. In agreement with the microarray data (Cernadas *et al.*, 2008), *CsCAF1* was preferentially induced in response to Xa infection at 24 and 48 h after bacterial inoculation (Fig. 1A). On the other hand, *CsLOB1* was highly and predominantly induced by Xc infection, whereas *PR1* was similarly up‐regulated by both pathogens at 24 and 48 h post‐infection (Fig. 1B,C). These results thus confirm our previous data (Abe and Benedetti, 2016; Cernadas *et al.*, 2008; Pereira *et al.*, 2014) and show that *CsCAF1* expression correlates with the defence response triggered by Xa in sweet orange plants.

### CsCAF1 is a Mg‐dependent deadenylase of the RNase D superfamily

**Figure 2:**
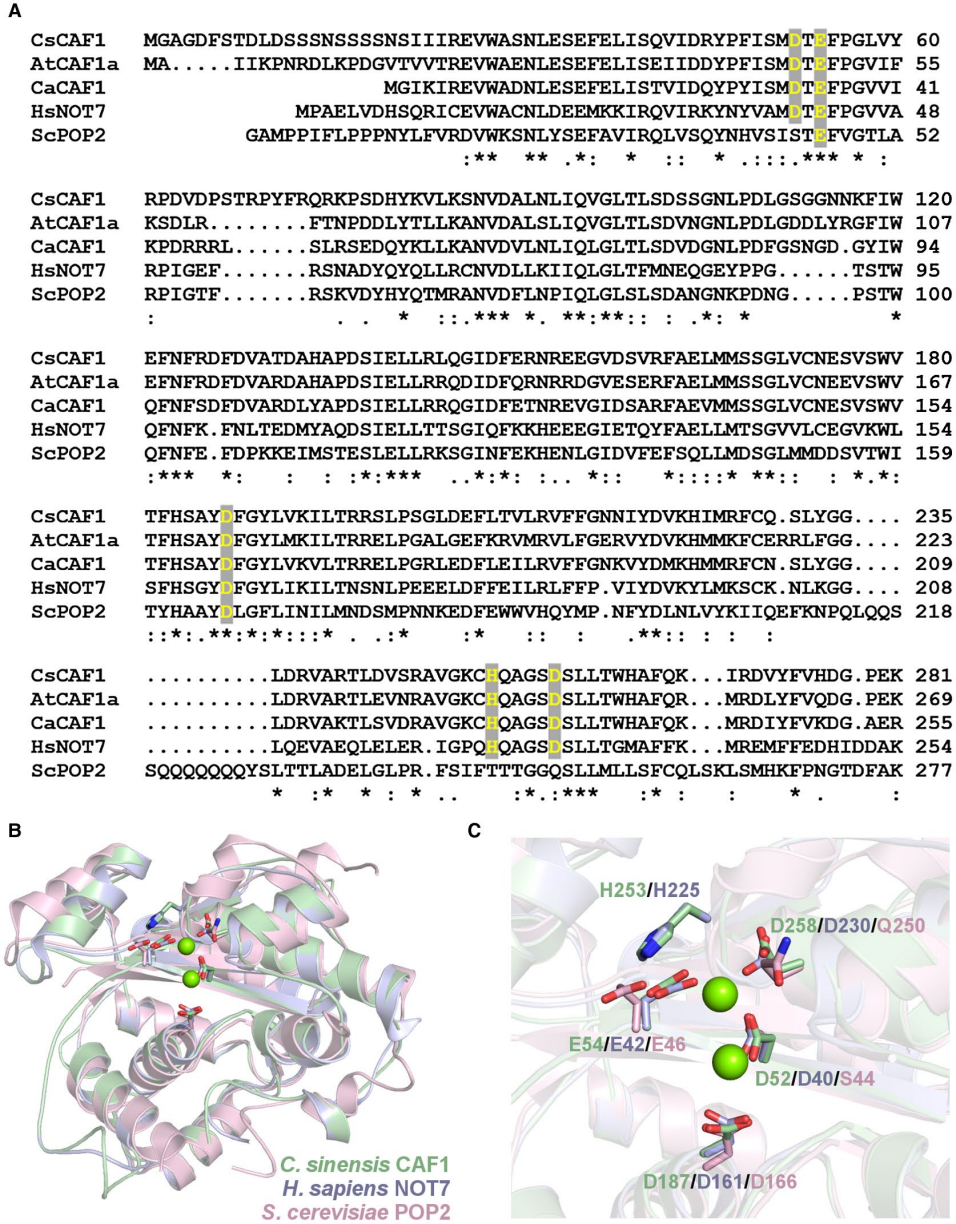
CsCAF1 is structurally related to plant, yeast and mammalian proteins belonging to the RNase D superfamily. (A) Protein sequence alignment performed with Clustal Omega showing that CsCAF1 is related to *C. annum* CaCAF1 (NP_001312000.1), *A. thaliana* AtCAF1a (OAP03728.1), *H. sapiens* HsNOT7 (AAP97145.1) and the RNase domain of *S. cerevisiae* ScPOP2 (EDN62858.1). The consensus DEDDh motif, characteristic of the RNase D superfamily, is conserved in CsCAF1 (residues shown in yellow). (B) Superposition of the crystal structures of yeast POP2 (PDB code 1UOC, pink) and human NOT7 (PDB code 4GMJ_B, blue) with the structural model of CsCAF1 (green) generated by SWISS‐MODEL using the human NOT7 structure as the search template. A close inspection of the active site of the proteins shows that the DEEDh motif in CsCAF1, comprising residues D52, E54, D187, H253 and D258, is structurally conserved relative to POP2 and NOT7. These resides are thought to also coordinate two magnesium ions, as shown in the NOT7 structure (green spheres).

CsCAF1 is closely related to *C. annum* CaCAF1 and * Arabidopsis* AtCAF1a (Liang *et al.*, 2009; Sarowar *et al.*, 2007), sharing up to 75% sequence identity with them. CsCAF1 is also 47% identical to human CAF1, known as NOT7 (Horiuchi *et al.*, 2009), and 34% identical to the RNase domain of yeast POP2 (Thore *et al.*, 2003) (Fig. 2A). Structural modelling studies suggest that CsCAF1 has the same protein fold as human NOT7 and yeast POP2, and that the consensus DEDDh motif of the RNase D superfamily, comprising residues D52, E54, D187, H253 and D258, is structurally conserved in CsCAF1, relative to the crystal structures of POP2 and NOT7 (Fig. 2B,C).

**Figure 3:**
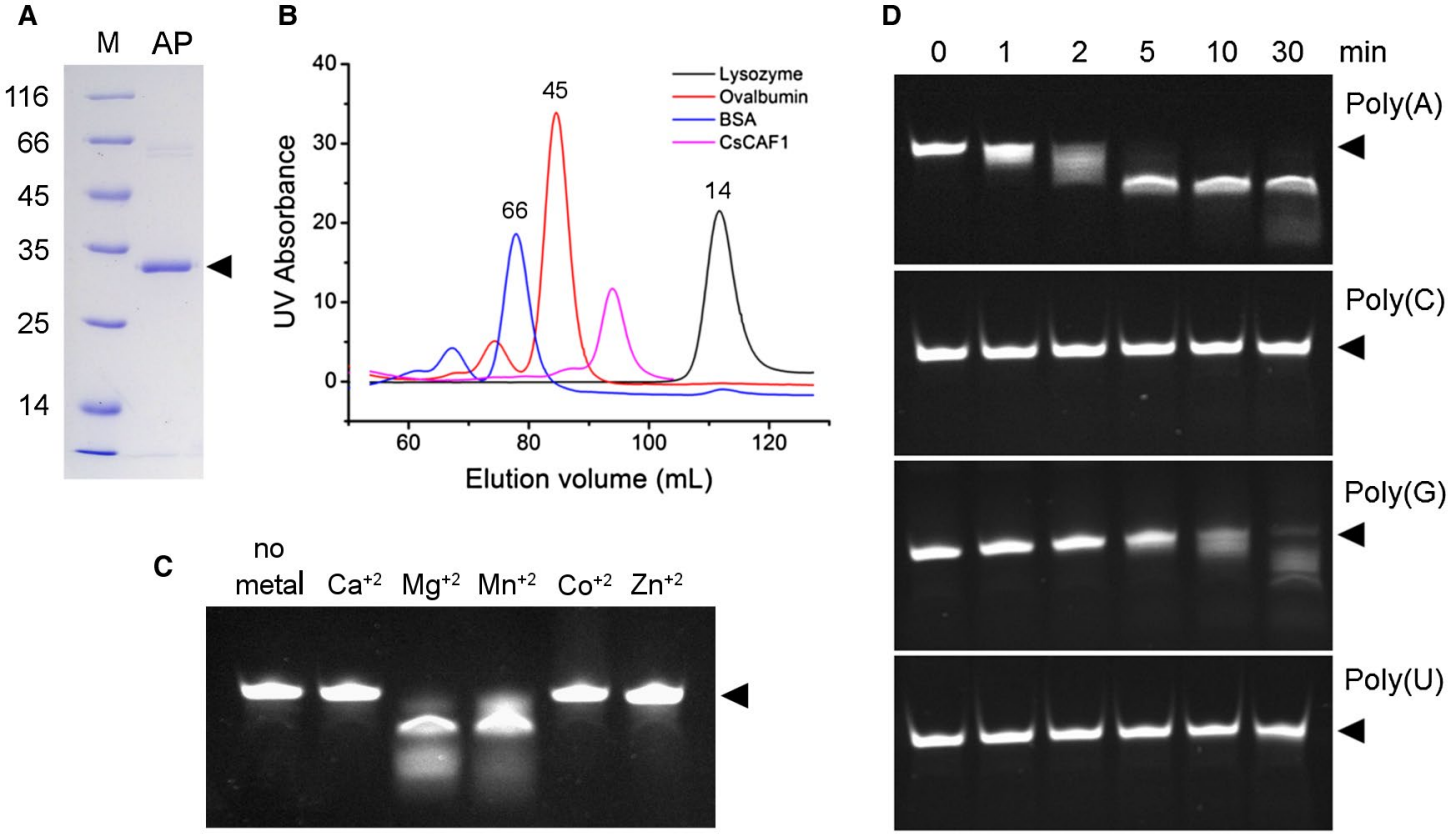
CsCAF1 is a Mg‐dependent deadenylase. (A) Denaturing polyacrylamide gel showing the recombinant 6xHis‐CsCAF1 purified by affinity chromatography. M and AP denote molecular ladder and affinity purified CsCAF1, respectively. CsCAF1 migrates with an apparent molecular weight of 32 kDa (arrowhead). (B) Size exclusion chromatography showing that CsCAF1 elutes with an estimated molecular mass of 31 kDa, relative to the elution volumes of Bovine Serum Albumin (BSA) (66 kDa), ovalbumin (45 kDa) and lysozyme (14 kDa). (C) Agarose gel showing that the deadenylase activity of recombinant CsCAF1 upon the poly(A) probe (arrowhead) is dependent on Mg^+2^ ions. A small deadenylase activity is observed in the presence of Mn^+2^, but no activity is detected in the presence of Ca^+2^ or Co^+2^ ions. (D) The 3ʹ–5ʹ exonuclease activity of CsCAF1 is selective to poly(A). Although a weaker RNase activity was detected on the poly(G) RNA probe 30 min after the addition of CsCAF1 to the reaction mixture, no RNase activity was observed in the presence of the poly(U) or poly(C) RNA probes. Arrowheads indicate undigested RNA probes.

To know whether CsCAF1 would exhibit a 3′–5′ exoribonuclease activity, recombinant CsCAF1 was produced and purified by affinity and size exclusion chromatography (Fig. 3A). CsCAF1 migrated with an apparent molecular mass of ~32 kDa in denaturing polyacrylamide gels and eluted with an estimated molecular mass of 31 kDa in size exclusion chromatography (Fig. 3B), suggesting that it is a monomer in solution. Purified CsCAF1 was incubated with the single‐strand poly(A) RNA probe (5′‐GACUGACUAAAAAAA‐3′) labelled with fluorescein (FITC) at its 5′ end (Horiuchi *et al.*, 2009). The exoribonuclease assays were performed in the presence of metal salts, since metal ions were shown to be required for NOT7 deadenylase activity (Horiuchi *et al.*, 2009). The results show that, like NOT7, CsCAF1 presents 3′–5′ exoribonuclease activity in the presence of magnesium, but not calcium, cobalt or zinc ions. A residual deadenylase activity was also observed in the presence of manganese (Fig. 3C).

Next, we tested the specificity of CsCAF1 towards different RNA molecules and found that CsCAF1 shows selective and stronger RNase activity towards poly(A) RNA. No RNase activity was observed with the poly(U) (5′‐GACUGACUUUUUUUU‐3′) or poly(C) (5′‐GACUGACUCCCCCCC‐3′) probes. Nevertheless, a weaker RNase activity was detected when the poly(G) probe (5′‐GACUGACUGGGGGGG‐3′) was used as substrate (Fig. 3D). These results thus show that CsCAF1 preferentially degrades poly(A) tails.

Because in yeast *caf1* deletion mutants exhibit sensitivity to high doses of caffeine (Hata *et al.*, 1998; Liu *et al.*, 1997), we decided to test whether CsCAF1 could serve as a functional homologue of the yeast protein by complementing this phenotype. However, CsCAF1 did not complement the yeast *caf1* mutant regardless of the caffeine dose used (Fig. S1, see Supporting Information). The low degree of homology to POP2 (Fig. 2A) and the fact that POP2 has a distinct SEDQt active site might explain the lack of CsCAF1 complementation in yeast.

### CsCAF1 interacts with PthAs and proteins associated with the CCR4‐NOT complex

**Figure 4:**
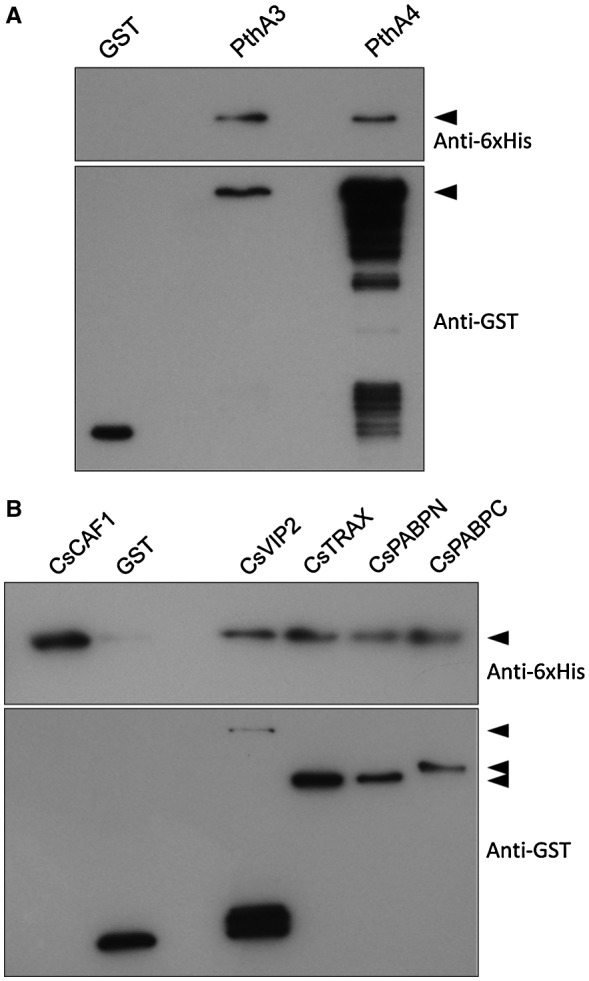
CsCAF1 interacts with Xc TAL effectors and with proteins associated with the citrus CCR4‐NOT complex. (A) GST‐pulldown assay showing that CsCAF1 interacts with the *X. citri* TAL effectors PthA3 and PthA4. The 6xHis‐CsCAF1 and GST‐PthAs detected by the anti‐6×His and anti‐GST antibodies, respectively, are indicated by arrowheads. (B) GST‐pulldown assay showing that CsCAF1 interacts with the citrus proteins CsTRAX and CsPABPN, and with the C‐terminal region of CsPABPC (aa 327 to 652). A weaker interaction is observed with CsVIP2. The 6×His‐CsCAF1 and GST‐fusions detected by the anti‐6×His and anti‐GST antibodies, respectively, are indicated by arrowheads.

We found previously that the Xc effector proteins PthA3 and PthA4, required to induce cankers on citrus (Abe and Benedetti, 2016), interacted with several citrus proteins implicated in mRNA stabilization and translational control, including the CsVIP2 (VirE2‐interacting protein 2), a NOT2 homologue, translin‐associated factor X (CsTRAX), and the poly(A)‐binding proteins CsPABPN and CsPABPC (de Souza *et al.*, 2012). Because these proteins interact with each other and are homologous to the mammalian proteins associated with the miRISC/CCR4‐NOT complex, they are thought to represent components of the citrus CCR4‐NOT complex (de Souza *et al.*, 2012). To verify this and examine whether CsCAF1 could also interact with PthAs, the recombinant CsVIP2, CsTRAX, CsPABPN, CsPABPC, PthA3 and PthA4 fused to GST were purified and used in GST‐pulldown assays as baits (Fig. S2, see Supporting Information). We found that, in addition to PthA3 and PthA4 (Fig. 4A), CsCAF1 interacted with CsTRAX, CsPABPN, CsPABPC and, to a lesser extent, CsVIP2 (Fig. 4B), suggesting that it is a component of the citrus CCR4‐NOT complex.

### PthA4 inhibits CsCAF1 deadenylase activity and stabilizes the CsLOB1 mRNA

**Figure 5:**
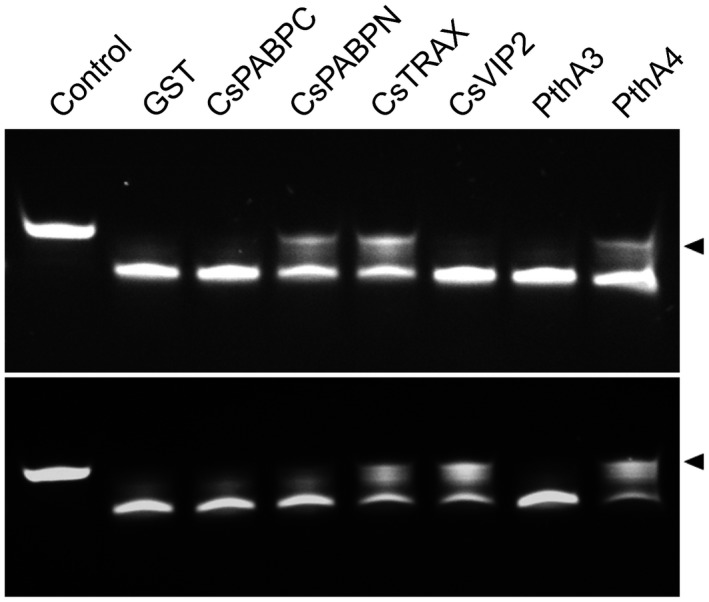
CsCAF1 deadenylase activity is inhibited by PthA4, CsTRAX and CsVIP2. The* in vitro* CsCAF1 deadenylase activity was assayed by mixing the GST‐fusion proteins (CsPABPC, CsPABPN, CsTRAX, CsVIP2, PthA3 and PthA4), or GST alone as control, with the poly(A) RNA probe before the addition of CsCAF1 to the reaction mixture (upper panel). Conversely, the deadenylase reaction was assayed by mixing the GST‐fusion proteins with CsCAF1 before the addition of the RNA probe (lower panel). The reaction was stopped 5 min after the incubation with all the reagents. Control represents a reaction mixture with no CsCAF1.

The interaction of CsCAF1 with PthAs, CsPABPC, CsPABPN, CsTRAX and CsVIP2 (Fig. 4) led us to test whether any of these protein interactors could influence the CsCAF1 deadenylase activity *in vitro*. We found that when PthAs or the citrus proteins were incubated with the poly(A) probe before the addition of CsCAF1 to the reaction mixture, CsPABPN, CsTRAX and PthA4 inhibited the deadenylase activity in the first 5 min of reaction. However, when the interacting proteins were incubated with CsCAF1 before the addition of the poly(A) probe, we noticed that, in addition to CsTRAX and PthA4, CsVIP2, but not CsPABPN, also inhibited the deadenylase activity of CsCAF1 (Fig. 5). These results suggest that CsTRAX, CsVIP2 and PthA4 do not merely compete with CsCAF1 for RNA binding, as it appears to be the case with CsPABPN. Additionally, although PthA3 interacted with CsCAF1 in GST‐pulldown assays (Fig. 4), this effector did not inhibit CsCAF1 deadenylase activity *in vitro* (Fig. 5).

CAF1 proteins are thought to promote mRNA degradation in response to a sudden increase in mRNA transcription as a way to balance the mRNA levels (Liang *et al.*, 2009). Because *CsLOB1* is highly and directly transcribed by PthA4 and rapidly accumulates during canker development (Abe and Benedetti, 2016; Hu *et al.*, 2014; Pereira *et al.*, 2014), we thought that CsCAF1 might target the *CsLOB1* mRNA to counteract its massive production upon PthA4 induction. However, given that PthA4 inhibited CsCAF1 deadenylase activity *in vitro*, we hypothesized that PthA4 could also inhibit CsCAF1‐dependent deadenylation of *CsLOB1* to stabilize this message. This idea is consistent with the fact that PthA4 interacts with several citrus proteins implicated in mRNA stabilization (Domingues *et al.*, 2015; de Souza *et al.*, 2012) and that the increased expression of CsCAF1 in citrus leaves infiltrated with the citrus canker bacteria correlates with a decrease in *CsLOB1* expression (Fig. 1A).

**Figure 6:**
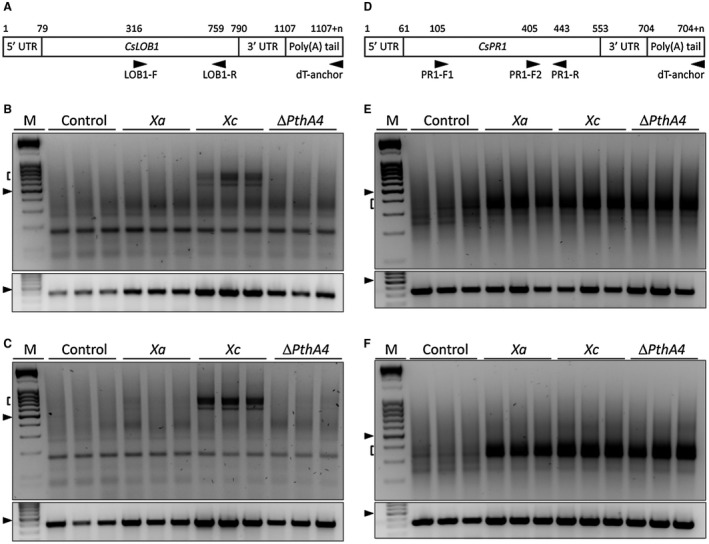
The *CsLOB1* mRNA poly(A) tail is stabilized in Xc‐infected leaves. (A) Schematic representation of the *CsLOB1* transcript showing the positions of the oligonucleotides used to detect the poly(A) tail (LOB1‐F and dT‐anchor) and the ~440 bp fragment of the *CsLOB1* coding region (LOB1‐F and LOB1‐R). The PAT assays for *CsLOB1* performed with RNA samples isolated from citrus leaves at 24 and 48 h after bacterial or water infiltration (control) are shown in the upper panels in (B) and (C), respectively. The corresponding amplification reactions for the *CsLOB1* coding region are shown in the bottom panels in B and C, respectively. (D) Schematic representation of the *CsPR1* transcript showing the positions of the oligonucleotides used to detect the poly(A) tail (PR1‐F2 and dT‐anchor) and the ~ 340 bp fragment of the *CsPR1* coding region (PR1‐F1 and PR1‐R). The PAT assays for the *CsPR1* transcript performed with the same RNA samples are shown in the upper panels in (E) (24 h post‐inoculation) and (F) (48 h post‐inoculation), whereas the corresponding amplification reactions for the *CsPR1* coding region are shown in the bottom panels in E and F, respectively. Arrowheads in B, C, E and F indicate the 0.5 kb marker band, whereas brackets indicate the lengths of the poly(A) tails. RNA samples from three independent citrus leaves infiltrated with water, Xa, Xc or the Xc *pthA4*‐delition mutant were examined.

To examine this, the poly(A) tail length of the *CsLOB1* mRNA in citrus leaves infiltrated with water, Xa, Xc or the Xc *pthA4*‐deletion mutant, was analysed by the PAT assay (Salles and Strickland, 1999). We found polyadenylated *CsLOB1* mRNA bands more abundantly in Xc‐infected leaves compared to leaves inoculated with Xa or the Xc *pthA4*‐deletion mutant, both at 24 and 48 h after bacterial infiltration. On the other hand, a fragment of the *CsLOB1* coding region was detected in all the leaf samples inspected, including those inoculated with the *pthA4*‐deletion mutant (Fig. 6A–C). The detection of the *CsLOB1* coding region in all examined leaves is in line with the results shown in Fig. 1 and with previous expression data showing that *CsLOB1* is also induced by Xa or the Xc *pthA4*‐deletion mutant (Abe and Benedetti, 2016; Pereira *et al.*, 2014).

The same experiment was performed to evaluate the poly(A) tail length of the mRNA encoded by the citrus *CsPR1* gene, which, although highly induced by Xc and Xa (Fig. 1 and Cernadas *et al.*, 2008), is not a direct target of PthA4 (Pereira *et al.*, 2014). In contrast to *CsLOB1*, the *CsPR1* mRNA poly(A) tail was strongly detected in all the leaves that had been infiltrated with the citrus canker pathogens, including the *pthA4*‐deletion mutant (Fig. 6D–F). Together, these results show that the *CsLOB1* mRNA poly(A) tail is specifically protected in Xc‐infected leaves during initial canker development, suggesting that PthA4 not only increases *CsLOB1* transcription but also stabilizes this message.

### A CsCAF1 inhibitor promotes canker development and inhibits PthA4‐dependent transcription

**Figure 7:**
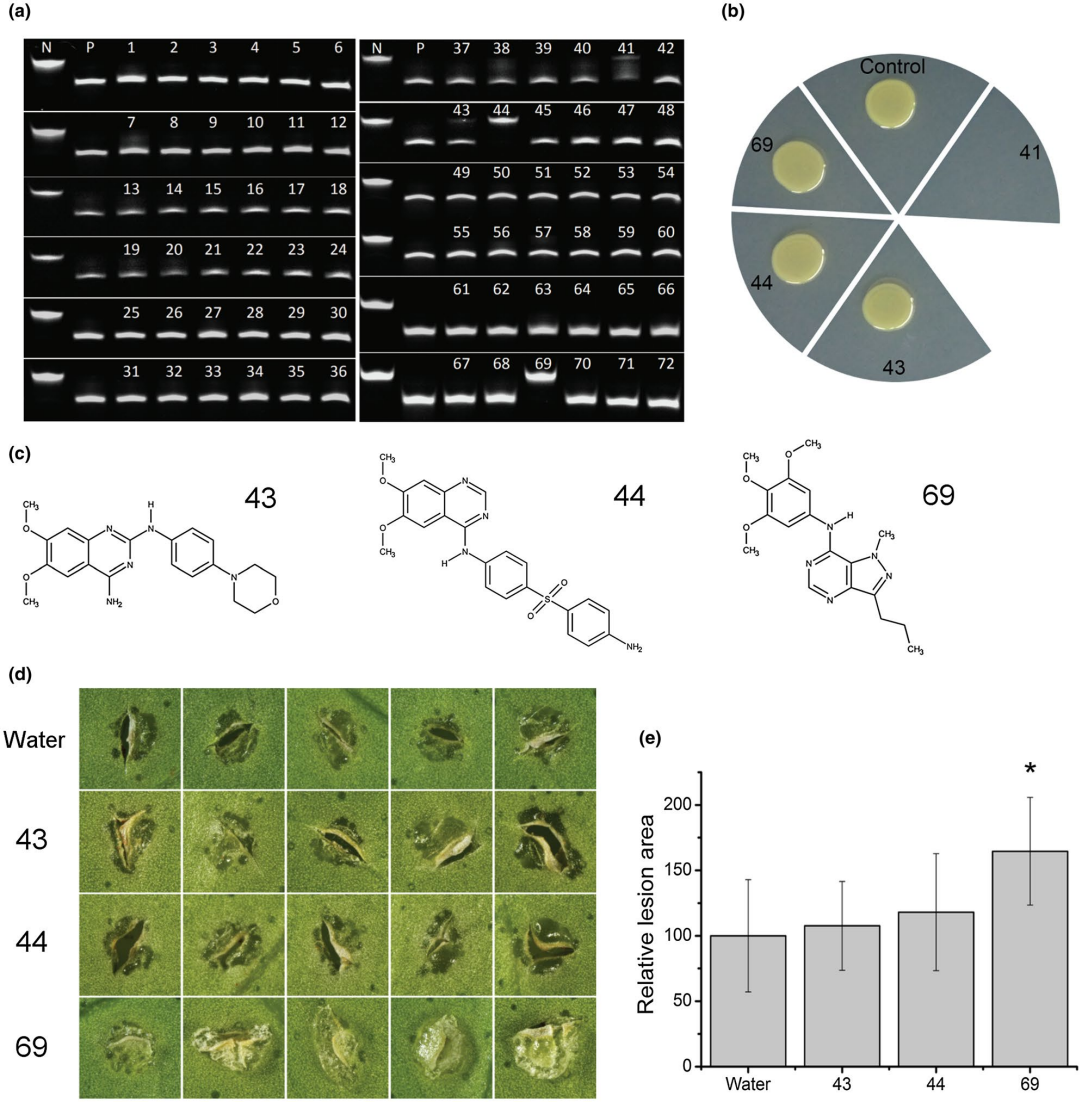
A CsCAF1 inhibitor enhanced canker formation in citrus leaves. (A)* In vitro* CsCAF1 deadenylase activity assayed in the presence of pyrazolo[4,3‐d]‐pyrimidine and quinazoline derivatives. Compounds 41, 43, 44 and 69 inhibited, to different degrees, the CsCAF1 deadenylase activity at 80 µM concentration. Control reactions include samples with no CsCAF1 (N) or with CsCAF1 purified by affinity chromatography (P). (B) At 160 µM, compounds 43, 44 and 69 did not inhibited the growth of Xc in culture medium. (C) Chemical structures of compounds 43, 44 and 69. (D) Representative images of canker lesions developed in sweet orange leaves infected with Xc 10 days after bacterial inoculation. Cell hypertrophy and hyperplasia was significantly enhanced in leaves treated with compound 69 compared to compounds 43, 44 or water control. (E) Relative lesion area showing that canker lesions formed in leaves treated with compound 69 were significantly enlarged compared to those developed in control leaves. Values are the means of measurements performed in 48 independent canker lesions developed in four citrus leaves. Error bars represent the standard deviations and the asterisk denotes statistically significant difference at the 0.05 level, relative to control (water).

To search for small molecules that could serve as inhibitors of CsCAF1, a series of pyrazolo[4,3‐d]‐pyrimidine and quinazoline derivatives were synthetized and verified by nuclear magnetic resonance and mass spectrometry analysis, as reported previously (Rocco *et al.*, 2004). The isolated molecules were tested for inhibition of CsCAF1 deadenylase activity *in vitro*. From a group of 72 test compounds, four compounds (41, 43, 44 and 69) were selected based on their capacity to inhibit CsCAF1 deadenylase activity at 80 µM concentration (Fig. 7A). These molecules were also tested for their ability to alter canker development in citrus leaves inoculated with Xc. Because compound 41 completely inhibited Xc growth in culture medium (Fig. 7B), it was not tested in citrus leaves and will be described elsewhere. Notably, we found that compound 69, an pyrazolo[4,3‐d]‐pyrimidine derivative that strongly inhibited CsCAF1 deadenylase activity *in vitro*, significantly enhanced canker formation in sweet orange leaves, compared to compounds 43 and 44 (Fig. 7A,C,D). Canker lesions developed in leaves treated with compound 69 were more enlarged and showed more epidermal rupture than those of untreated control leaves (Fig. 7D,E), indicating that compound 69 promotes cell division and growth induced by Xc.

These results led us to test whether compound 69 was stabilizing the *CsLOB1* mRNA. Surprisingly, we found that compound 69 did not apparently change the poly(A) tail length or stability of the *CsLOB1* and *CsPR1* transcripts, but significantly inhibited the PthA4‐dependent transcription of *CsLOB1* without affecting *CsPR1* transcription (Fig. S3, see Supporting Information). These results were confirmed by qPCR analyses which also show that compound 69 induced *CsCAF1* expression in Xc‐infected leaves, but repressed *CsCAF1* expression in leaves inoculated with the *pthA4*‐deletion mutant (Fig. S3, see Supporting Information). Because *CsCAF1* expression was also significantly enhanced in leaves infiltrated with the *pthA4*‐deletion mutant, the results suggest that PthA4 negatively regulates *CsCAF1* levels and supports the negative correlation observed between *CsCAF1* and PthA4‐dependent *CsLOB1* expression.

## Discussion

CAF1 has been extensively studied in yeast and animals, but its role in plant development and response to pathogens remains largely uncharacterized. Here we show that, similar to tomato and *Arabidopsis* CAF1, which play a role in pathogen defence (Lee *et al.*, 2004; Liang *et al.*, 2009; Sarowar *et al.*, 2007), CsCAF1 is also implicated in the resistance against citrus canker bacteria. For instance, increased CsCAF1 expression correlated with a decrease in the expression of *CsLOB1*, the major canker susceptibility gene induced by the Xc TAL effector PthA4 (Hu *et al.*, 2014). Moreover, an inhibitor of CsCAF1 deadenylase activity, compound 69, significantly enhanced canker development in citrus leaves, suggesting that CsCAF1 restricts plant cell growth induced by Xc.

The mechanism by which CsCAF1 would contribute to bacterial resistance or restrict canker development is unknown; however, the fact that PthA4 inhibited CsCAF1 deadenylase activity *in vitro* and CsCAF1 transcription *in vivo*, and that polyadenylated *CsLOB1* mRNA was more abundantly detected in citrus leaves infected with Xc than in leaves infected with Xc lacking *pthA4* suggests that CsCAF1 might play a role in the transcription and/or stability of citrus canker susceptibility genes induced by PthA4.

We showed previously that PthA4 interacted with components of the citrus CCR4‐NOT complex, including CsTRAX, CsPABPN, CsPABPC and CsVIP2 (de Souza *et al.*, 2012). Here, we show that CsCAF1 also interacts with these proteins, indicating that it is a component of the citrus CCR4‐NOT complex. It is thus possible that PthA4 could target the citrus CCR4‐NOT complex to enhance transcription and translation of disease susceptibility messages. This idea is supported by the fact that the CCR4‐NOT complex also interacts with Pol II to promote transcription elongation, particularly from arrested Pol II (Dutta *et al.*, 2015; Kruk *et al.*, 2011; Reese, 2013). Moreover, like PthA4, the * Arabidopsis* CsVIP2 homologue AtNOT2 binds the C‐terminal domain of Pol II and enhances transcription of the *CsLOB1* homologues *AtLOB‐1* and *AtLOB‐11* (Domingues *et al.*, 2012; Wang *et al.*, 2013). It should also be noted that PthA4 binds poly(U) RNA and is structurally related to PUF (pumelo and FBF) proteins, which recognize U‐rich sequences at the 3′‐end of mRNAs and are known to interact with CAF1 and recruit the CCR4‐NOT complex to modulate mRNA stability and translation (Goldstrohm *et al.*, 2006; Filipovska and Rackham, 2012; de Souza *et al.*, 2012; Van Etten *et al.*, 2012; Wang et al., 2018). Thus, given that the CCR4‐NOT complex is a macromolecular machine that connects transcription elongation to translation (Babbarwal *et al.*, 2014; Gupta *et al.*, 2016; Villanyi *et al.*, 2014; Webster *et al.*, 2018; Yi *et al.*, 2018), it seems reasonable to suggest that PthA4 could modulate its activity to enhance transcription and translation of citrus canker susceptibility genes.

Recombinant CsCAF1 showed specific activity towards poly(A) RNA and its deadenylase activity was dependent on magnesium ions. In this respect, CsCAF1 retains the major structural and functional properties of DEDDh nucleases. In fact, molecular modelling studies revealed that CsCAF1 is also structurally related to DEDDh nucleases that share no obvious sequence identity to it. This is the case of the human magnesium‐dependent poly(A)‐specific ribonuclease (PARN), which is also involved in poly(A) tail shortening (Körner and Wahle, 1997; Wu *et al.*, 2005). According to our structural models, CsCAF1 not only shows the same type of fold as human PARN, but also shares the main amino acid residues involved in RNA recognition and poly(A) specificity that were mapped in the crystal structure of PARN in complex with poly(A) RNA (Wu *et al.*, 2005). This indicates that CsCAF1 likely shows the same poly(A)‐binding mode as human PARN (Fig. S4, see Supporting Information). Moreover, as shown for the *Schizosaccharomyces pombe* Pop2p protein (Jonstrup *et al.*, 2007), the high degree of conservation of the DEDDh active site and divalent metal ion‐binding residues among these enzymes suggest that CsCAF1 would also display the same mechanism of 3′–5′ exonuclease cleavage as Pop2p and PARN.

Small molecules that inhibit CAF1 deadenylase activity have recently been reported for human CAF1 (Jadhav *et al.*, 2015; Maryati *et al.*, 2014, 2015; Zhang *et al.*, 2016). Because these inhibitors are not commercially available, we have synthesized a series of pyrazolo[4,3‐d]‐pyrimidine and quinazoline derivatives with the purpose of finding novel CAF1 inhibitors. Here we show that compound 69, an pyrazolo[4,3‐d]‐pyrimidine analogue, significantly inhibited CsCAF1 deadenylase activity *in vitro* while it promoted canker development in citrus leaves, indicating that CsCAF1 restricts cell growth in citrus. Although this result is consistent with the antitumour role played by CAF1‐Tob proteins in mammalian cells (Doidge *et al.*, 2012; Hosoda *et al.*, 2011), it contrasts with the findings that overexpression of *Capsicum annum* CAF1 led to enlarged leaf cells in pepper plants (Sarowar *et al.*, 2007). In addition, compound 69 significantly increased canker formation despite decreasing *CsLOB1* expression. This is, nevertheless, not a surprise since we have evidence suggesting that high *CsLOB1* expression alone is not sufficient to induce citrus canker in sweet orange plants (Abe and Benedetti, 2016). Unfortunately, despite several attempts, we were unable to transiently modulate CsCAF1 expression in citrus leaves by *Agrobacterium*‐mediated transformation (Jia and Wang, 2014). Therefore, the precise role of CsCAF1 in cell growth control, possibly involving transcription and translation of *CsLOB1* or other canker susceptibility genes, will require further study.

## Experimental Procedures

### Protein expression and purification

The cDNA corresponding to the *CsCAF1* gene was subcloned into pET28a for the expression of full‐length 6×His‐tagged CsCAF1 in *Escherichia coli* BL21 (DE3) cells. Bacterial cells were grown at 37 °C in LB medium containing kanamycin (50 µg/mL) to an OD_600_ ∼0.6, after which 0.1 mM isopropylthio‐β‐D‐galactoside (IPTG) was added to the culture. The cells were grown overnight at 18 °C, harvested by centrifugation and lysed by sonication in lysis buffer containing 20 mM Tris‐HCl, pH 8.0, 200 mM NaCl, 20% glycerol, 40 mM imidazole, 1.0 µg/mL lysozyme and 400 U DNase I (Sigma‐Aldrich, San Luis, Missouri, USA). The suspension was centrifuged to remove cell debris and recombinant CsCAF1 was purified from the soluble fraction in a cobalt HiTrap chelating HP column (GE Healthcare, Chicago, Illinois, USA). The column was washed with ten column volumes of lysis buffer and CsCAF1 was eluted with the same buffer containing 200 mM imidazole. CsCAF1 fractions, analysed by SDS‐PAGE, were concentrated, dialyzed against 20 mM Tris‐HCl, pH 8.0, 300 mM NaCl, 5% glycerol, and loaded on a Hi‐load 16/60 Superdex 200 column (GE Healthcare) pre‐equilibrated with the same buffer. CsCAF1 fractions eluted in a single peak were analysed by SDS‐PAGE and concentrated. For estimation of CsCAF1 molecular weight, protein samples were loaded on an analytical Superdex‐200 10/300 GL column, equilibrated with the same buffer and calibrated with a set of molecular weight standards (GE Healthcare). The elution volumes of the standards were used to generate a linear plot of the retention coefficients versus the logarithm of the molecular weight, which was used to estimate the molecular weight of CsCAF1.

GST and GST‐tagged CsVIP2, CsTRAX, CsPABPN, CsPABPC (residues 327 to 652), PthA3 and PthA4, described previously (de Souza *et al.*, 2012), were also expressed in *E. coli* BL21 cells upon induction with 0.1 mM IPTG. The cell pellets were suspended in phosphate‐buffered saline (PBS) containing 150 mM NaCl, lysozyme (1.0 µg/mL) and DNase I. After sonication and centrifugation, soluble fractions of GST‐tagged proteins were immobilized on glutathione resin (GE Healthcare) and unbound proteins were removed with four PBS washes. Elution was performed in PBS containing 7 mg/mL reduced glutathione. The quality of the purified proteins was analysed by SDS‐PAGE.

### GST pulldown assays

GST and the GST‐tagged proteins CsVIP2, CsTRAX, CsPABPN, CsPABPC, PthA3 and PthA4 were immobilized on separate glutathione resin columns and unbound proteins were removed with four PBS washes. Purified 6×His‐CsCAF1 was passed through each of the GST columns and the beads were washed four times with PBS to remove unbound proteins. Bound proteins were eluted with reduced glutathione and resolved on 12% SDS‐PAGE gels. Proteins were transferred onto polyvinylidene difluoride membranes, probed with the anti‐His (1:3000) and anti‐GST (1:3000) and detected by chemiluminescence (Thermofisher, Waltham, Massachusetts,USA).

### Deadenylase activity assay

To test the CsCAF1 exonuclease activity, the following RNA probes carrying a FITC tag at the 5′ position (Horiuchi *et al.*, 2009) were used: GACUGACUUUUUUUU, GACUGACUAAAAAAA, GACUGACUCCCCCCC and GACUGACUGGGGGGG. Each tagged RNA was incubated at 37 °C with purified CsCAF1 (0.5 µM) in 20 mM HEPES buffer, pH 7.4, containing 150 mM NaCl and 1 mM DTT, and supplemented with 2 mM MgCl_2_, CaCl_2_, MnCl_2_, CoCl_2_ or ZnCl_2_ for different time periods. The reactions were stopped by the addition of an equal volume of 37% (v/v) formamide. Ten microlitres of the reaction mixtures were loaded on denaturing DNA sequencing gels and the RNA probes were detected under UV‐light using a FITC filter. For CsCAF1 inhibition assays, 80 μM of the candidate inhibitors, or dimethyl sulfoxide (DMSO) as a control, were added to the reaction mixtures.

### Yeast complementation

The yeast *pop2* deletion mutant YI3945 and corresponding wild‐type strain BY4742 were obtained from the National BioResource Project (Japan). The mutant was transformed with the citrus *CsCAF1* gene cloned into the pESC‐URA vector (Agilent, Santa Clara, Califórnia, USA). For gene complementation assays, yeast cells were grown on SG medium plates without leucine, in the absence or presence of 0.2, 0.5 or 2 mM caffeine at 30 °C for 2 days.

### Gene expression analysis

*Xanthomonas citri* and *X. aurantifolii* pathotype C (Cernadas *et al.*, 2008) were grown on LB medium without NaCl (LBON), supplemented with 100 mg/L ampicillin, for 48 h at 28 °C. Single colonies were suspended in sterile water to an OD_600_ of 0.1, and the bacterial suspensions were used to infect sweet orange (*C. sinensis*) plants of cultivar Natal (Washington Navel) kept in the green house. Citrus leaves of similar age and size were infiltrated with the bacterial suspensions, or water as control, as described previously (Abe and Benedetti, 2016; Cernadas *et al.*, 2008). At 24 and 48 h after bacterial infiltration, leaf tissues were ground in liquid nitrogen and total RNA was extracted using Trizol (Invitrogen, Carlsbad, CA, USA). The quality and quantity of the RNA were verified by agarose gel. The RNA samples were treated with DNase I to remove traces of DNA and cDNAs were synthesized using the Maxima First Strand cDNA Synthesis kit (Fermentas, Waltham, MA, USA) according to the supplier's instructions. The cDNA samples were diluted and tested for specificity and amplification efficiency of the probed genes relative to the actin gene used as the endogenous control (Mafra *et al*., 2012). Three PCRs were performed for each gene studied and three biological replicates were analysed using the SYBR Green mix and the universal conditions of amplification provided by the 7500 System (Applied Biosystems, Foster City, CA, USA). The results were analysed by the 7500 System software (Applied Biosystems) using the relative quantification mode and expressed as the mean of nine amplification curves. Primers used for RT‐qPCR analysis include qPR1‐F GCAAGGTGTGTGGGCACTATAC, qPR1‐R ACCCAATGCGAACCGAATT, qCAF1‐F TCGTCGGGACTCGTCTGTAAC, qCAF1‐R AATCGTACGCGCTGTGGAA, qLOB1‐F TTTTCCACCAACCGAACCAT, qLOB1‐R TGATATTGCTAGCACCGAAGACTCT, qActin‐F CCCTTCCTCATGCCATTCTTC and qActin‐R CGGCTGTGGTGGTAAACATGT.

### Protein sequence alignment and molecular modelling

Protein sequence alignments were performed with Clustal Omega using default parameters, whereas a tridimensional model of CsCAF1 was generated with Swiss‐Model using the crystal structure of human CAF1 protein (PDB code 4GMJ_B) (Petit *et al.*, 2012) as the template model. Protein structures were aligned and visualized with PyMOL (Schrödinger, 2010).

### Synthesis of CAF1 inhibitors

CsCAF1 inhibitors were synthesized based on previous protocols with minor modifications (Rocco *et al.*, 2004). The syntheses were performed in single steps with the utilization of a thionyl chloride to obtain the 2‐chloro‐quinazoline, 4‐chloro‐quinazoline and 4‐chloro‐pyrazolo[4,3‐d]‐pyrimidine derivatives. The chlorine served as a leaving group to facilitate the nucleophilic aromatic substitution in the final synthesis step. This final step utilized the corresponding anilines and resulted in good yields. The protocols for the synthesis of compounds 43, 44 and 69 are detailed in the Supplementary Protocol S1. All compounds were dissolved in DMSO at 10 mM concentration and kept at –80 °C until use.

### In vivo CsCAF1 inhibition assay

*Xanthomonas citri* was grown as above and a bacterial suspension in sterile water (OD 600 nm = 0.1) was used to infect Natal leaves by pinprick inoculations (Soprano *et al*., 2017). One hour after bacterial inoculation, the leaves were detached and placed in recipients containing water supplemented with 160 µM of the inhibitor molecules or DMSO as control. The leaves were kept in a plant growth room with a 12/12 h day/night light period at 25 °C. Treatment solutions were replaced every 2 days. Canker pustules were photographed 8 days after bacterial inoculation using a Nikon SMZ18 stereomicroscope, and the area of the lesions was measured using ImageJ software.

For bacterial survival assay, the *X. citri* cells in the water suspension were treated with 160 µM of the inhibitor molecules, or DMSO as control, for 1 h at 28 °C, after which they were spotted onto a LBON medium and incubated at 28 °C for 16 h.

### Poly(A) tail length assay

The poly(A) tail length determination was performed using the PAT assay (Salles and Strickland, 1999). Total RNA was extracted from leaves infiltrated with water, *X. citri* or *X. aurantifolii* for 24 or 48 h, as described above. For cDNA construction, 2 µg of RNA, diluted in 5 µL diethyl pyrocarbonate (DEPC)‐treated water, were mixed with 2 µL (20 ng) oligo dT_18_ and the reaction was incubated at 65 °C for 10 min. The tubes were transferred to 42 °C and 13 µL of a pre‐warmed reverse transcriptase master mix containing 4 µL 5× ReverseAid H minus first‐strand cDNA synthesis reaction buffer, 2 µL 0.1 M DTT, 2 µL 10 mM dNTP mixture, 1 µL 10 mM ATP, 2 µL DEPC‐treated water and 2 µL T4 DNA ligase (10 U) were added to the reaction mixtures. After incubation at 42 °C, 1 µL (200 ng) oligo dT‐anchor (GACTCGAGTCGACATCGACCCTTTTTTTTTTTTTTTTTT) was added and each reaction was mixed and incubated at 12 °C for 2 h. After incubation, the reactions were warmed at 42 °C for 2 min and 1 µL of ReverseAid H minus first‐strand cDNA synthesis enzyme was added and incubated at 42 °C for 1 h. The reverse transcriptase and ligase enzymes were heat inactivated by incubation at 65 °C for 20 min and the cDNA samples were used for the PCRs. Detection of the poly(A) tail length was carried out using oligos LOB1‐F1 CCACCAACCGAACCATACAAGTTCACC or PR1‐F2 (GCAAGGTGTGTGGGCACTATAC) in combination with oligo dT‐anchor, and the amplified fragments were resolved by electrophoresis on a 2.0% agarose gel stained with ethidium bromide. Control PCRs for the amplifications of *CsLOB1* and *PR1* coding regions were performed with the pair of oligonucleotides LOB1‐F1 and LOB1‐R (AGAGGCTCCCAAGCTGATCCA), and PR1‐F1 (TCCCATGCACAAGACTCACC) and PR1‐R (ACCCAATGCGAACCGAATT), respectively.

## Supporting information


**Fig. S1** Functional complementation assay of the yeast pop2 mutant showing that CSCAF1 does not complement the caffeine sensitivity phenotype of the yeast mutant in SD medium containing 0.2 mM, 0.5 mM or 2.0 mM caffeine.

**Fig. S2** Ten percent polyacrylamide SDS PAGE gel of the recombinant proteins 6×His CsCAF1, GST and GST fusions CsPABPC, CsPABPN, CsVIP2, CsTRAX, PthA3 and PthA4, purified by affinity chromatography. The arrows indicate the corresponding protein bands with the expected molecular size. The molecular mass ruler is indicated on the left.

**Fig. S3** The CsCAF1 inhibitor  compound 69  modulates the expression of *CsCAF1*, *CsLOB1* but not *CsPR1* in citrus leaves. (A) PAT assay showing that compound 69 significantly inhibited the accumulation of polyadenylated *CsLOB1* but not *CsPR1* transcripts in Xc infected plants only, at 48 h post‐ bacterial inoculation. (B) RT qPCR analyses showing that compound 69 significantly inhibited the PthA4‐dependent expression of *CsLOB1* but not *CsPR1* in Xc infected leaves, corroborating the PAT assay data depicted in panel A. Conversely, compound 69 induced the expression of *CsCAF1* and *CsLOB1*, but not * CsPR1* , in noninfected leaves. The expression levels of * CsCAF1* was also significantly increased in leaves inoculated with the * pthA4*  deletion mutant, which suggests that PthA4 represses *CsCAF1* in citrus leaves. This PthA4‐dependent repression of *CsCAF1* was inhibited by compound 69.

**Fig. S4** CsCAF1 shares the same protein fold and poly(A) binding mode as human PARN. (A) Superposition of the crystal structure of human PARN (PDB code 2A1R, grey) with the structural model of CsCAF1 (green) generated by SWISS MODEL using the human NOT7 structure as the search template. CsCAF1 shows the same type of protein fold as human PARN despite sharing low sequence identity to PARN. (B) Close view of the active site of the proteins showing the conservation of the amino acid residues (sticks) involved in RNA recognition between PARN and CsCAF1. The magnesium ions suggested to participate in the hydrolyses of the RNA phosphodiester bond are shown as green spheres.
