# Supplementary material for: Role of the Citrus sinensis RNA deadenylase CsCAF1 in citrus canker resistance
Source: Mol Plant Pathol. 2019 May 21;20(8):1105–18. doi: 10.1111/mpp.12815 (PMC6640180; doi:10.1111/mpp.12815)
Supplement: Supplementary file 1 — Fig. S1 Functional complementation assay of the yeast pop2 mutant showing that CSCAF1 does not complement the caffeine sensitivity phenotype of the yeast mutant in SD medium containing 0.2 mM, 0.5 mM or 2.0 mM caffeine. [file MPP-20-1105-s001.pdf]

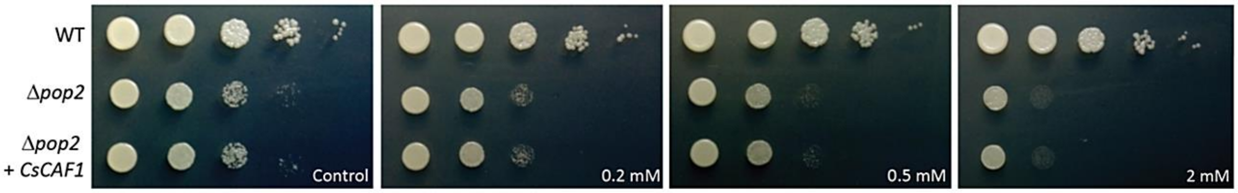

Fig. S1. Functional complementation assay of the yeast *pop2* mutant showing that CSCAF1 does not complement the caffeine sensitivity phenotype of the yeast mutant in SD medium containing 0.2 mM, 0.5 mM or 2.0 mM caffeine.
