# Supplementary material for: Role of the Citrus sinensis RNA deadenylase CsCAF1 in citrus canker resistance
Source: Mol Plant Pathol. 2019 May 21;20(8):1105–18. doi: 10.1111/mpp.12815 (PMC6640180; doi:10.1111/mpp.12815)
Supplement: Supplementary file 2 — Fig. S2 Ten percent polyacrylamide SDS PAGE gel of the recombinant proteins 6×His CsCAF1, GST and GST fusions CsPABPC, CsPABPN, CsVIP2, CsTRAX, PthA3 and PthA4, purified by affinity chromatography. The arrows indicate the corresponding protein bands with the expected molecular size. The molecular mass ruler is indicated on the left. [file MPP-20-1105-s002.docx]

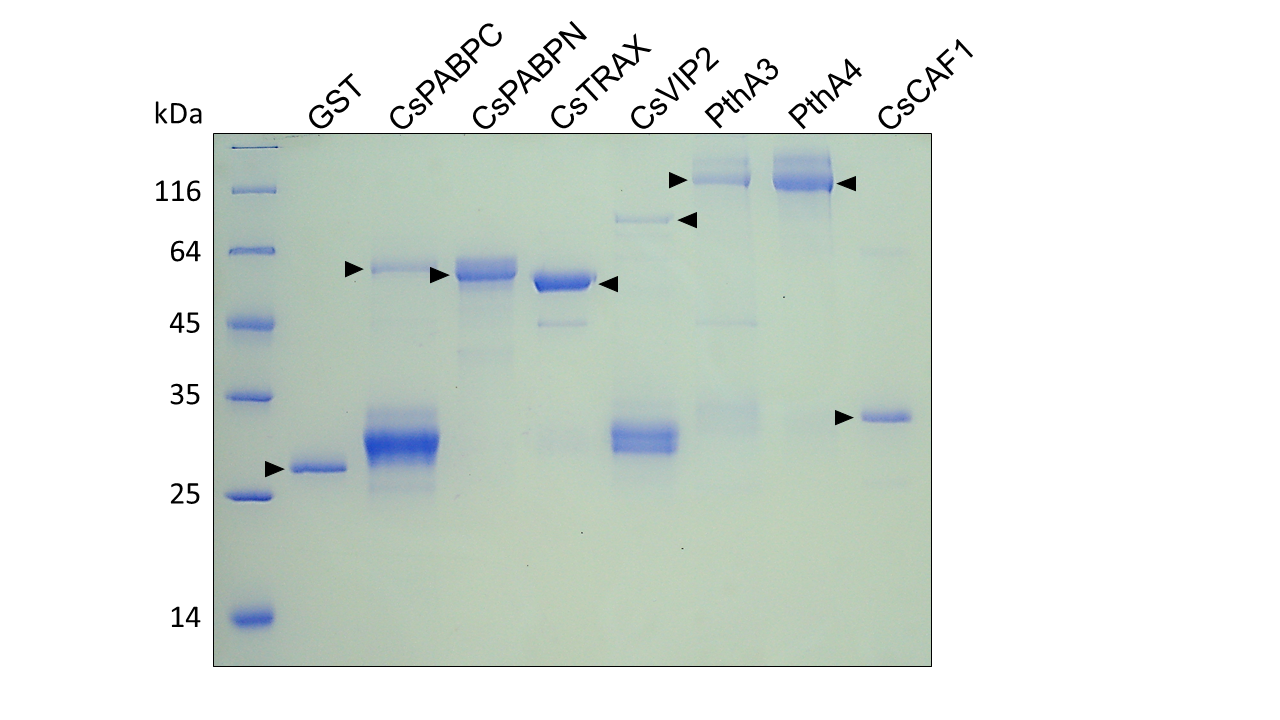


**Fig. S2**. Ten percent polyacrylamide SDS-PAGE gel of the recombinant proteins 6xHis-CsCAF1, GST and GST-fusions CsPABPC, CsPABPN, CsVIP2, CsTRAX, PthA3 and PthA4, purified by affinity chromatography. The arrows indicate the corresponding protein bands with the expected molecular size. The molecular mass ruler is indicated on the left.
