# Supplementary material for: Role of the Citrus sinensis RNA deadenylase CsCAF1 in citrus canker resistance
Source: Mol Plant Pathol. 2019 May 21;20(8):1105–18. doi: 10.1111/mpp.12815 (PMC6640180; doi:10.1111/mpp.12815)
Supplement: Supplementary file 3 — Fig. S3 The CsCAF1 inhibitor compound 69 modulates the expression of CsCAF1, CsLOB1 but not CsPR1 in citrus leaves. (A) PAT assay showing that compound 69 significantly inhibited the accumulation of polyadenylated CsLOB1 but not CsPR1 transcripts in Xc infected plants only, at 48 h post‐ bacterial inoculation. (B) RT qPCR analyses showing that compound 69 significantly inhibited the PthA4‐dependent expression of CsLOB1 but not CsPR1 in Xc infected leaves, corroborating the PAT assay data depicted in panel A. Conversely, compound 69 induced the expression of CsCAF1 and CsLOB1, but not CsPR1 , in noninfected leaves. The expression levels of CsCAF1 was also significantly increased in leaves inoculated with the pthA4 deletion mutant, which suggests that PthA4 represses CsCAF1 in citrus leaves. This PthA4‐dependent repression of CsCAF1 was inhibited by compound 69. [file MPP-20-1105-s003.docx]

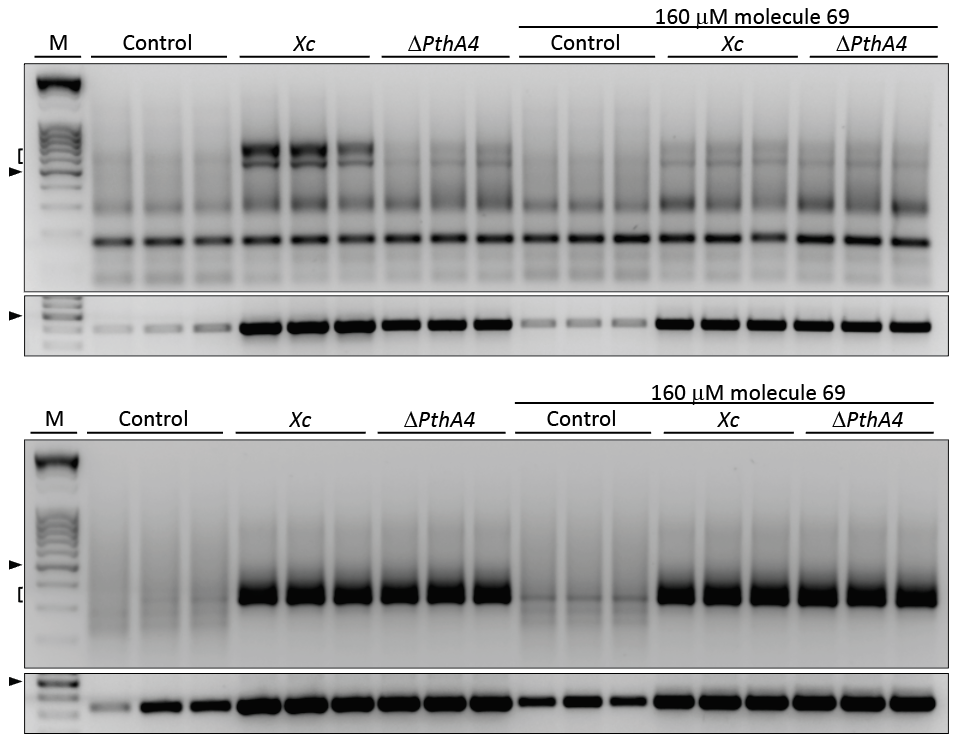
A Untreated Compound 69

*CsLOB1*

*CsPR1*


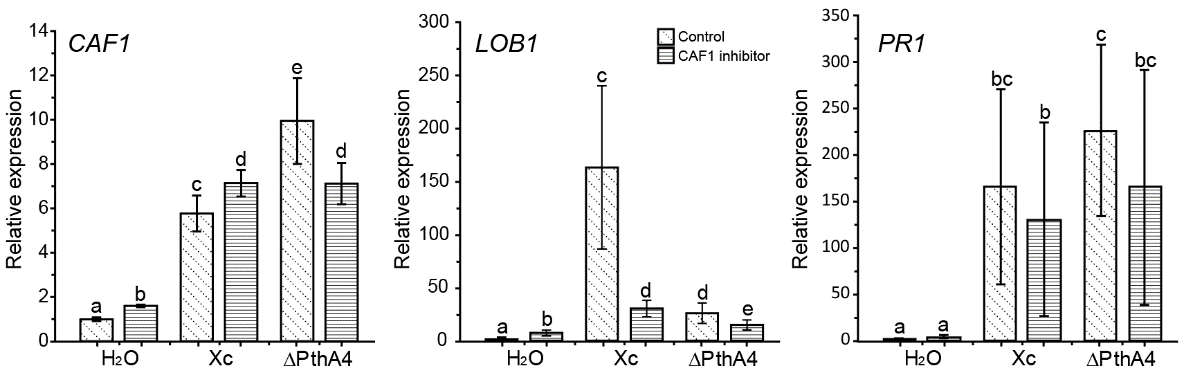
B

**Fig. S3**. The CsCAF1 inhibitor ‘compound 69’ modulates the expression of *CsCAF1*, *CsLOB1* but not *CsPR1* in citrus leaves. (A) PAT assay showing that compound 69 significantly inhibited the accumulation of polyadenylated *CsLOB1* but not *CsPR1* transcripts in Xc-infected plants only, at 48 h post bacterial inoculation. (B) RT-qPCR analyses showing that compound 69 significantly inhibited the PthA4-dependent expression of *CsLOB1* but not *CsPR1* in Xc-infected leaves, corroborating the PAT assay data depicted in panel A. Conversely, compound 69 induced the expression of *CsCAF1* and *CsLOB1*, but not *CsPR1*, in non-infected leaves. The expression levels of *CsCAF1* was also significantly increased in leaves inoculated with the *pthA4*-deletion mutant, which suggests that PthA4 represses *CsCAF1* in citrus leaves. This PthA4-dependent repression of *CsCAF1* was inhibited by compound 69.
