# Supplementary material for: Role of the Citrus sinensis RNA deadenylase CsCAF1 in citrus canker resistance
Source: Mol Plant Pathol. 2019 May 21;20(8):1105–18. doi: 10.1111/mpp.12815 (PMC6640180; doi:10.1111/mpp.12815)
Supplement: Supplementary file 4 — Fig. S4 CsCAF1 shares the same protein fold and poly(A) binding mode as human PARN. (A) Superposition of the crystal structure of human PARN (PDB code 2A1R, grey) with the structural model of CsCAF1 (green) generated by SWISS MODEL using the human NOT7 structure as the search template. CsCAF1 shows the same type of protein fold as human PARN despite sharing low sequence identity to PARN. (B) Close view of the active site of the proteins showing the conservation of the amino acid residues (sticks) involved in RNA recognition between PARN and CsCAF1. The magnesium ions suggested to participate in the hydrolyses of the RNA phosphodiester bond are shown as green spheres. [file MPP-20-1105-s004.docx]

A B


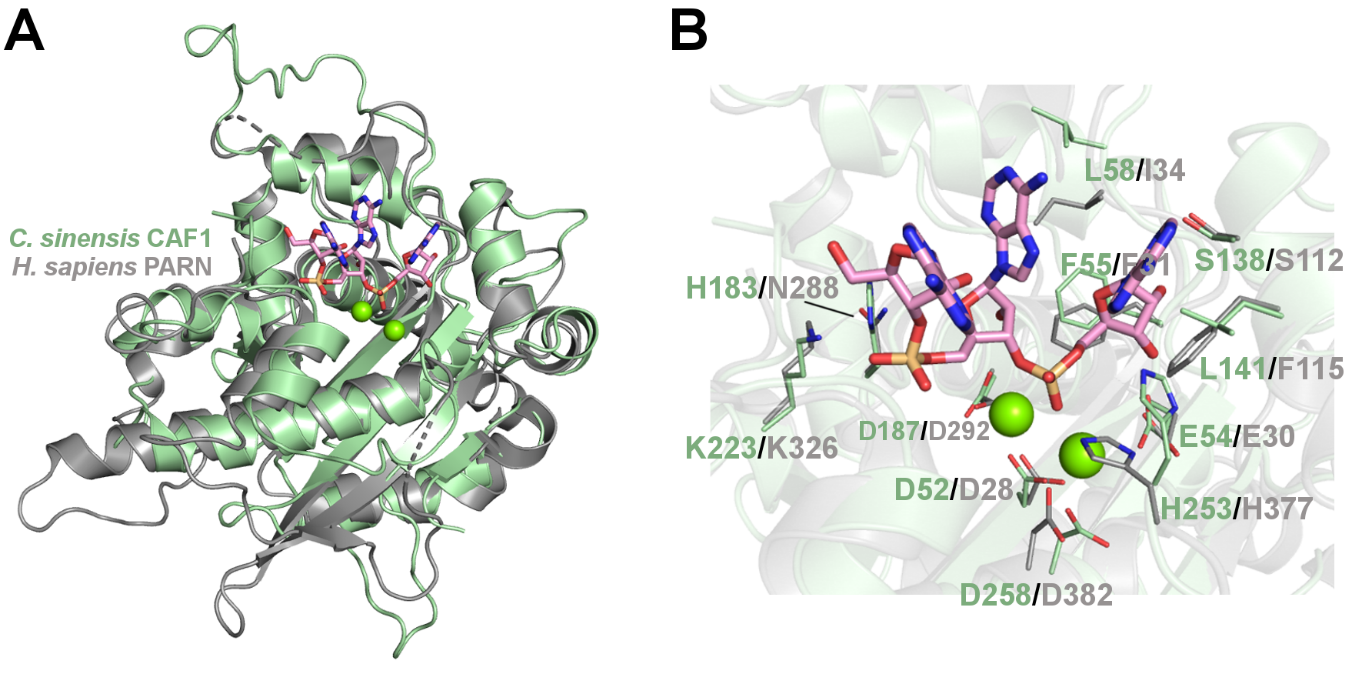


**Fig. S4**. CsCAF1 shares the same protein fold and poly(A)-binding mode as human PARN. (A) Superposition of the crystal structure of human PARN (PDB code 2A1R, gray) with the structural model of CsCAF1 (green) generated by SWISS-MODEL using the human NOT7 structure as the search template. CsCAF1 shows the same type of protein fold as human PARN despite sharing low sequence identity to PARN. (B) Close view of the active site of the proteins showing the conservation of the amino acid residues (sticks) involved in RNA recognition between PARN and CsCAF1. The magnesium ions suggested to participate in the hydrolyses of the RNA phosphodiester bond are shown as green spheres.
